# Supplementary material for: Footprint evidence of early hominin locomotor diversity at Laetoli, Tanzania
Source: Nature. 2021 Dec 1;600(7889):468–71. doi: 10.1038/s41586-021-04187-7 (PMC8674131; doi:10.1038/s41586-021-04187-7)
Supplement: Supplementary file 1 — This file contains Supplementary Discussion (Laetoli A rediscovery and description; Agriotherium) and Supplementary Methods (human cross-stepping footprint experiment; proportional toe depth measurement). [file 41586_2021_4187_MOESM1_ESM.pdf]

nature portfolio

https://doi.org/10.1038/s41586-021-04187-7

Supplementary information

Footprint evidence of early hominin locomotor diversity at Laetoli, Tanzania

In the format provided by the authors and unedited

Supplementary Discussion

Laetoli A Rediscovery and Description

The footprint Site A in Locality 7 was accidentally discovered on July 24, 1976, by Andrew Hill and Kay Behrensmeyer while they were famously engaged in an elephant dung fight with David (Johan) Western, Dorothy (Doty) Dechant, and Philip Leakey. In September of that year, Peter Jones and Philip Leakey identified five consecutive bipedal footprints at the A site. In June of 2019, we attempted to relocate the Site A bipedal footprint trail.

Leakey and Harris[3] noted that the central part of Site A was subject to heavy erosion and that only a small portion (24 m^2) of the footprint tuff where the bipedal tracks were found remained. Given forty years of additional erosive action, we assumed the rest of the central portion of the A site, including the bipedal tracks would be gone. Nevertheless, we used the exquisitely detailed footprint maps located in Leakey and Harris[3] to approximate the location of the bipedal tracks. Using the maps, we estimated that the southern hook in the eastern edge of the upper part of Site A to be 54.5 meters from the bipedal footprint trail. On Wednesday June 19, 2019, B. Maley and L. Fannin cleared enough overburden in this area to locate juvenile proboscidean footprints that are—according to the Leakey and Harris[3] maps—about three meters east of the bipedal tracks. We measured to a spot in which K. Fabian dug and was able to find footprint A3 and a 155 mm long, 70 mm wide pothole located 200 mm west of the hominin(?) footprint. We used a hard bristled brush to remove the infill and then a tongue depressor to clear any additional debris from the track. With this certainty that the trail had not eroded, we temporarily covered the footprint with sand and returned two days later to resume the excavation. From June 21-24, we continued the excavation, clearing all the overburden and

rediscovering all five of the original A site footprints. We extended the excavation south of the first footprint by 0.87 meters and east 0.54 meters. The latter was done in case the footprint maker had changed direction from the west to the north prior to making the A1 track. This area of the footprint ash was poorly laminated and difficult to separate. Nevertheless, we found no evidence of additional bipedal footprints. On June 25, photographs were taken such that photogrammetry could be undertaken, after which the tracks were reburied and covered with a cairn of stones so that they can be easily relocated by future researchers, and by conservators interested in the long-term preservation of these footprints.

The five footprints are angled 340 degrees north, northwest. The second and third footprints (A2 and A3) are the best preserved, though A1, A4, and A5 are detectable. All are described below.

<u>A1</u> A1 is a poorly preserved left footprint. The proximal edge of the heel is clearly delineated as is the distal edge of the footprint making the 165 mm foot length a reliable measurement. The preserved heel width is a minimum of 42 mm. Medially, there are two likely lagomorph tracks that obscure the medial border of the foot preventing a reliable measurement of the forefoot. There is a raised ridge of hardened sediment positioned 57 mm from the distal end of the heel separating the hindfoot from the forefoot. Two cracks in the footprint tuff crisscross the footprint. Step length between A1 and A2 is 325 mm.

<u>A2</u> A2 is a right footprint preserving 160 mm from the proximal rim of the heel to the tip of the hallux. The hallux impression is clear and well defined, measuring 36 mm mediolaterally. The heel impression is 65 mm wide; the forefoot is 88 mm wide. As in A1, there is a raised ridge of hardened ash separating the heel from the forefoot. A crack in the footprint tuff layer runs proximomedially to distolaterally through the forefoot.

There is a large (58 mm ML, 48 mm PD) piece of tuff adhered to the footprint in the middle portion of the forefoot. Efforts to remove it failed. Step length between A2 and A3 is 310 mm.

A3 A3 is a well-preserved left footprint. It measures 160 mm from the proximal rim of the heel to the distal rim of the toes. Interiorly, from the tip of the scalloped, semi-circular impression of the second digit to the base of the heel, the footprint is 125 mm long. The heel impression from rim-to-rim is 65 mm wide. Interiorly, the heel is 47 mm wide. Distal to the rim of the heel impression by approximately 42 mm is a raised ridge of hardened sediment that is 14 mm thick proximodistally. The forefoot is 97 mm wide. The hallux impression is clear and 30 mm wide mediolaterally. It projects distally more than the rest of the forefoot, making it the longest of the digits. It is separated from the lateral digits by a raised mound of hardened matrix that is 13 mm wide (between the first and second toes), continues to widen proximally to 37 mm and extends 66 mm proximally into the medial forefoot region. This gravel filled matrix is not laminated and is continuous with the rest of the footprint, suggesting to us that it was part of the landscape at the time this hominin pressed its foot into the Laetoli ash. Lateral to this matrix is the scalloped impression of the second digit, 17 mm wide. Faint impressions delineated the lateral three toes. To the east, 154 mm from the heel of footprint A3 is a large proboscidean track that is approximately 450 mm wide and 400 mm long. Step length between A3 and A4 is 335 mm.

A4 A4 is a right footprint. The medial portion of the heel is preserved but laterally, there is raised sediment obscuring the edge of the heel. It is a minimum of 46 mm wide. During excavation, chisel impressions were inadvertently made into the medial edge of the heel

rim. The medial edge of the footprint easily crumbles and is ill-defined. Nevertheless, the forefoot width could be estimated based on what was preserved: 90 mm. Distally, the footprint meets a crumbling, laminated ash-layer. Exploratory excavation of this distal area failed to locate any evidence of toe impressions. What is preserved of the entire footprint is 150 mm long, in which case only 10 mm of foot length is missing (from what is known of the other footprints). Step length between A4 and A5 is 310 mm.

A5 A5 is a poorly preserved left footprint located at the edge of the eroded part of Site A central. Proximally, there is a narrow heel impression, but the middle and distal portion is amorphous, precluding any measurements and detailed anatomical information.

The measurements reported above differ slightly from those reported by Tuttle[4] and require some explanation. The average step length (N=4) is 320 mm, which is nearly identical to the 323 mm reported in Tuttle[4]. Additionally, our calculation of step width (N=1; -24.8 mm) is almost the same as Tuttle[4] (-27.0 mm). Attempts to calculate step width for the other footprints besides the A2-A3 transition were prone to error and not included though they qualitatively appear to us to be similar to the -24.8 mm we report. For foot length, forefoot width and heel width, we measured from rim-to-rim of the footprint. For foot length, we calculate an average of 161.7 mm, longer than the 141.2 mm reported in Tuttle[4]. Measuring from the tip of the hallux to the deepest, most proximal impression of the heel on the 3D scan of A3, we could replicate the ~141 mm measurement, meaning that the differences in measurement may be the result of using internal versus external footprint landmarks. Since the external are more clearly delineated, we prefer that approach. Furthermore, this approach would not impact ratios and qualitative observations, which are the basis for our interpretations in this study. Despite that, we are unable to replicate the heel width or forefoot width measurements in Tuttle[4]. Our average heel width of

64.9 mm is taken on the external rim of the heel and is similar to the 59.2 cm reported in Tuttle[4]. Internally, we get a smaller number of ~47 mm, though these measurements are subject to error given the curved outline of the heel and the sloping rims of the heel impression of track A3. Our forefoot width of 91.7 mm (N=3) is significantly wider than the 75.8 mm reported in Tuttle[4]. The narrowest, most internal part of the forefoot of track A3 is ~85 mm. Some of the different values reported here are almost certainly a function of a more thorough excavation of the footprints in 2019 compared to 1977 when they were not fully cleaned of their matrix infill[1,2,4,5,12].

Agriotherium

The only sub-Saharan Pliocene ursid currently known is *Agriotherium*. The first appearance of this genus in Africa comes from the 5.54-5.77 Ma Asa Koma Member of the Adu-Asa Formation, Middle Awash, Ethiopia[58]. Most of our understanding of *Agriotherium* derives from over 300 fossils from 14 *Agriotherium africanum* individuals from the late Miocene (~5 Ma) site of Langebaanweg, South Africa[59]. Eastern African *Agriotherium* fossils have been reported from the 4.5-5.0 Ma Nkondo (Uganda) and Sinda (DR Congo) formations[60]. These fossils, along with similarly aged material from the Mabaget formation, Tugen Hills, Kenya, have been identified as a distinct species, *Agriotherium aecuatorialis*[61]. *Agriotherium* sp. fossils have been reported from 4.4 Ma deposits at Aramis, Ethiopia[62,63], and the 3.5-3.8 Ma Woranso-Mille, Ethiopia site[64]—the latter of particular note since it overlaps in time with the footprint tuff at Laetoli. Furthermore, Werdelin and Lewis[65] report ursid fossils from the Denen Dora and Kada Hadar members of Hadar, Ethiopia, Tulu Bor member of Koobi Fora, Kenya and the lower Lomekwi, West Turkana, Kenya with 2.9 Ma as the date of last occurrence for sub-Saharan bears. *Agriotherium* fossils have not been reported from Kanapoi, Kenya[66], nor have any yet been found at Laetoli, Tanzania[17]. We caution, however, that although ursid fossils are not yet

known from Laetoli, *Agriotherium* was present in similarly aged sites in the African Rift Valley and may still have been present there.

The ~5.0 Ma South African site of Langabaanweg preserves the most postcranial material from *Agriotherium*[59] and the only published foot bones. Compared with modern ursids, *Agriotherium* is exceptionally large, the largest individuals estimated to have reached 540 kg[67], exceeding the size of a modern polar bear. This fossil bear possessed relatively long legs, and its foot bones are described as generally ursid-like, indicating that modern bear footprints should serve as a reasonable substitute for *Agriotherium* footprints (see below). There are some minor differences in relative metatarsal lengths but none that would lead one to expect hominin-looking footprints from the *Agriotherium* foot.

A 6.8 Ma ursid trackway from Coffee Ranch, Texas has been assigned to *Platykopus stuartjohnstoni*, an ursid possibly synonymous with *Agriotherium schneideri*, also known from this late Miocene locality[68]. The authors note that the fossil foot material of *Agriotherium* from Langabaanweg, South Africa is, "of the right shape and proportions to have been those of the trackmaker of *Platykopus stuartjohnstoni*." The authors draw attention to the plantigrady foot impression, but also note that the anterior extent of the digits form an arc in which the longest digit is the third and there is a clear separation between impressions made by the digits and by the ball of the foot.

While bear tracks—including (possibly) *Agriotherium*—possess anterior arcs in which the center of the foot is the longest, the Laetoli A3 footprint is mostly squared-off and, if anything, tapered towards what we interpret as the lateral side of the foot as occurs in hominin feet. Furthermore, while bear tracks—including those possibly made by *Agriotherium*—leave a

discernible cleft between the ball of the foot and the digits, the Laetoli A3 track leaves no such impression and instead there is a gradation between the ball of the foot and the toes as can be observed in the Laetoli G and S footprints. These differences between footprints produced by ursids and hominids have also been noted in the context of the Trachilos footprints from the Miocene of Crete[69,70].

Supplementary Methods

Human cross-stepping footprint experiments

Additional experiments were carried out by K.G.H. and E.M.W.-H. at Chatham University to further evaluate the ways in which cross-stepping kinematics influence human footprint sizes and morphologies. These experiments allowed us to better understand whether cross-stepping kinematics alone might be responsible for the observed differences between the Laetoli tracks at Site A and those from Sites G and S (i.e., to assess the likelihood that the Site A tracks could have been created by members of the same hominin species as represented at Sites G and S, but while they engaged in cross-stepping rather than maintaining their normal walking stride).

A wooden container measuring approximately 5 m long, 60 cm wide, and 7.5 cm deep was constructed and filled with sand at a depth of approximately 45 mm, following other experiments designed to resemble the sedimentary conditions of the volcanic ash in which the 3.66 Ma Laetoli footprints were created[37,52]. Sand was of heterogeneous grain size, as previous experiments have also shown that grain size heterogeneity has no effect on gross track

morphology[52,63]. A video camera was positioned perpendicular to the trackway, about 4.5 m from its center and at a height of 1 m, to record each trial from a lateral view.

In accordance with protocols approved by the Chatham University IRB, 10 habitually shod adults were recruited and gave their informed consent to participate in these experiments. we recruited a convenience sample from our university campus that was mixed with respect to age and gender. Our convenience sample included 10 adults between 19 and 48 years old, with 6 female, 3 male, and 1 non-binary recruited from the Chatham University campus. Neither age nor gender was expected to affect the hypotheses being tested (how footprint morphology changes due to cross-stepping). Anthropometric measurements including height, weight, hip height (height of greater trochanter), foot lengths (heel to hallux), and foot widths (first to fifth metatarsal heads) were measured from each subject. These measurements were taken using a tape measure (height, hip height, foot length), digital scale (weight), and digital sliding calipers (foot width). Each subject walked several times across the sand trackway to become comfortable moving across it. Once they were acclimated, subjects completed a total of 10 trials in which they walked barefoot from one end of the trackway to the other thereby creating footprints. For five trials, subjects used their normal, self-selected, comfortable walking gait. For the other five trials, they were instructed to walk using a cross-stepping gait (walking with a negative stride width), as has been inferred for the Site A hominin trackway at Laetoli. This inferred walking pattern was first demonstrated by the experimenters, and then subjects took several practice strides until they were comfortable walking in this manner. After practice, subjects completed five trials in which they produced a cross-stepping trackway during each trial. Following each trial, a centrally located footprint was selected for focused analysis. The lengths of the steps that bracketed that footprint were measured, as were the length and width of the stride. Between 25

and 30 photographs were taken of the focal track (typically 25, but up to 30 if the topography was more complex and required additional angles for full coverage), in order to create a 3-D scan using photogrammetry. These photographs were taken with multiple scales adjacent to the footprint, using a Canon 5D Mark III 22.3-Megapixel camera fitted with a 50 mm prime lens (Canon, Melville, NY, USA). Once these data were gathered, the sand was smoothed to its original state and the subjects moved on to the next walking trial.

Following experiments, Agisoft Metashape software (v.1.7.3; St. Petersburg, Russia) was used to generate scaled 3-D models of the focal tracks from each experimental trial. DigTrace Pro software (v.1.8.1)[53] was then used to generate 3-D models of an average normal walking track and an average cross-stepping track for each subject. Original models were cropped to include only a small amount of surrounding substrate, rotated such that their principal plane was orthogonal to vertical, mirrored if necessary (all models were oriented as right footprints), and then registered to the track within a given subset that had the clearest outline shape.

Once exported from DigTrace, average tracks were converted from point clouds to polygonal meshes using Geomagic Wrap 2021 (3D Systems; Rock Hill, SC, USA). Within Geomagic Wrap, linear measurements of the length and forefoot width of each track model were taken. The average differences in footprint lengths and widths between the two conditions were calculated, to understand whether and to what extent the different kinematic strategies influenced these footprint dimensions. Also, in Geomagic Wrap, regional depths were extracted from each of the 14 regions of interest that were measured in unshod human footprint experiments[42], such that these tracks could be run through the same multivariate distance analysis as the human, chimpanzee, and Laetoli samples, as presented in the main text. The multivariate distances, and

probabilities of sampling tracks similar to each subject's normal and cross-stepping tracks (from within the much broader unshod human track sample) were directly compared.

For cross-stepping trials, ratios between stride width and step length averaged -10.40 percent and ranged from -29.92 to 1.53, thereby falling near the observed values for Laetoli A (Extended Data Fig. 5b). Comparisons of linear measurements showed that, on average, a subject's mean cross-stepping footprint was 0.57 cm shorter and 0.11 cm wider than their mean normal walking footprint (range from 0.22 to 1.10 cm shorter, and from 0.32 cm narrower to 0.74 cm wider). These differences are relatively subtle and are likely a consequence of the different trajectories by which the foot enters and leaves the substrate. The foot would be expected to move primarily in the sagittal plane during a normal walking gait but would enter and leave the substrate at more oblique angles while cross-stepping.

Comparisons of internal footprint topology demonstrate that cross-stepping human footprints do not differ significantly from those produced by normal human walking gaits. As described above, experimental subjects walked with step width/step length ratios that essentially mirrored the pattern observed for the Laetoli A trackway (Extended Data Table 1). When doing so, they produced footprints whose average morphologies were statistically indistinguishable from the unshod modern human comparative sample (Extended Data Fig. 7). This further demonstrates the improbability that the Laetoli A tracks were produced by an individual with the same combination of foot anatomy and function as represented by the Laetoli G or S tracks.

Proportional Toe Depth Measurement

The proportional toe depth ratio was measured modeled off the methods in Raichlen and Gordon[41]. The individual footprint digital elevation models (DEM) for A2 and A3, which had

been previously adjusted to “level” the ground surface (see above), were analyzed for local toe and heel depth in ArcGIS (v. 10.7) using the "3D Analyst" toolbar. A line was created across the DEM that included the ground on both sides and passed through the lowest toe and heel depths. This line was used to extract a "profile graph" across these points from which the lowest elevation could be extracted for the toe and heel areas. The proportional toe depth measurement was calculated based on Raichlen and Gordon[41].

The proportional toe depth for Laetoli A2 was -0.258 (toe depth of 11.85, heel depth of 21.45). The proportional toe depth for Laetoli A3 was -0.123 (toe depth of 14.0, heel depth of 18.6). These values overlap with the lowest calculations from Laetoli S in Raichlen and Gordon[41] but are distinct from those found for Laetoli G1. The average proportional toe depth for Laetoli A (-0.191) is lower than the means observed for all other groups but falls within the lower end of the range of variation for modern humans walking with an extended limb gait and outside the range exhibited by humans moving with bent hip and bent knee posture.

Reference List:

58. Haile-Selassie, Y. & Howell, F. C. in *Ardipithecus kadabba. Late Miocene Evidence From the Middle Awash* (eds Haile-Selassie, Y. & WoldeGabriel, G.) (Univ. California Press, 2009)
59. Hendey, Q. B. *Agriotherium* (Mammalia, Ursidae) from Langebaanweg, South Africa, and relationship of this genus. *Ann. S. Afr. Mus*, **81** 1–222 (1980).
60. Yasui, K., Kunimatsu, Y., Yuga, N., Bajope, B. & Ishida, H. Fossil mammals from the Neogene strata in the Sinda basin, Eastern Zaire. *Afr. Study Monogr*. **Suppl. 17**, 87–107 (1992).

61. Morales, J., Pickford, M. & Soria, D. Carnivores from the Late Miocene and basal Pliocene of the Tugen Hills, Kenya. *Rev. de la Soc. Geol. de España* **18**, 19–61 (2005).

62. WoldeGabriel, G., et al Ecological and temporal placement of early Pliocene hominids at Aramis, Ethiopia. *Nature* **371**, 330–333 (1994).

63. White, T. D., et al Macrovertebrate paleontology and the Pliocene habitat of *Ardipithecus ramidus*. *Science* **326**, 67–93 (2009).

64. Haile-Selassie, Y., Deino, A., Saylor, B., Umer, M. & Latimer, B. Preliminary geology and paleontology of new hominid-bearing Pliocene localities in the center Afar region of Ethiopia. *J. Anthropol. Sci.* **115**, 215–222 (2007).

65. Werdelin, L., and Lewis, M. E. Plio-Pleistocene Carnivora of eastern Africa: species richness and turnover patterns. *Zool. J. Linnean Soc.* **144**, 121–144 (2005).

66. Werdelin, L., and Lewis, M. E. A contextual review of the Carnivora of Kanapoi. *J. Hum. Evol.* **140**, 102334 (2020).

67. Sorkin, B. Ecomorphology of the giant short-faced bears *Agriotherium* and *Arctodus*. *Hist. Biol.* **18**, 1–20 (2006).

68. Lucas, S. G. & Schultz, G. E. Miocene vertebrate footprints from the Texas Panhandle. *N. M. Mus. Nat. Hist. Sci. Bull.* **42**, 177–183 (2007).

69. Gierliński, G. D., et al Possible hominin footprints from the late Miocene (c. 5.7 Ma) of Crete? *Proc. Geol. Assoc.* **128**, 697–710 (2017).

70. Crompton, R. H. Making the case for possible hominin footprints from the Late Miocene (c. 5.7 Ma) of Crete? *Proc. Geol. Assoc.* **128**, 692–693 (2017).
