# Supplementary material for: Footprint evidence of early hominin locomotor diversity at Laetoli, Tanzania
Source: Nature. 2021 Dec 1;600(7889):468–71. doi: 10.1038/s41586-021-04187-7 (PMC8674131; doi:10.1038/s41586-021-04187-7)
Supplement: Supplementary file 2 — Reporting Summary [file 41586_2021_4187_MOESM2_ESM.pdf]

nature portfolio

Corresponding author(s): Ellison J. McNutt

Last updated by author(s): Sep 23, 2021

# Reporting Summary

Nature Portfolio wishes to improve the reproducibility of the work that we publish. This form provides structure for consistency and transparency in reporting. For further information on Nature Portfolio policies, see our Editorial Policies and the Editorial Policy Checklist.

## Statistics

For all statistical analyses, confirm that the following items are present in the figure legend, table legend, main text, or Methods section.

| n/a | Confirmed | |
|---|---|---|
| ☐ | ☒ | The exact sample size ($n$) for each experimental group/condition, given as a discrete number and unit of measurement |
| ☐ | ☒ | A statement on whether measurements were taken from distinct samples or whether the same sample was measured repeatedly |
| ☐ | ☒ | The statistical test(s) used AND whether they are one- or two-sided<br>*Only common tests should be described solely by name; describe more complex techniques in the Methods section.* |
| ☒ | ☐ | A description of all covariates tested |
| ☒ | ☐ | A description of any assumptions or corrections, such as tests of normality and adjustment for multiple comparisons |
| ☐ | ☒ | A full description of the statistical parameters including central tendency (e.g. means) or other basic estimates (e.g. regression coefficient) AND variation (e.g. standard deviation) or associated estimates of uncertainty (e.g. confidence intervals) |
| ☐ | ☒ | For null hypothesis testing, the test statistic (e.g. $F$, $t$, $r$) with confidence intervals, effect sizes, degrees of freedom and $P$ value noted<br>*Give P values as exact values whenever suitable.* |
| ☒ | ☐ | For Bayesian analysis, information on the choice of priors and Markov chain Monte Carlo settings |
| ☒ | ☐ | For hierarchical and complex designs, identification of the appropriate level for tests and full reporting of outcomes |
| ☒ | ☐ | Estimates of effect sizes (e.g. Cohen's $d$, Pearson's $r$), indicating how they were calculated |

*Our web collection on statistics for biologists contains articles on many of the points above.*

## Software and code

Policy information about availability of computer code

| | |
|---|---|
| Data collection | ImageJ (v. 1.49), Teckscan Footmat Research (v. 7.10), Agisoft Metashape (v. 1.4.4 and v. 1.7.1), Autodesk Meshmixer (v. 3.5.474) |
| Data analysis | R (v. 3.6.1 and v. 4.0.3), Excel (v. 2102), Ultimaker Cura (v. 4.8.0), Cloud Compare (v. 2.11.3), SAGA GIS (v. 7.3.0), ArcGIS (v. 10.6.1), MaxTRAQLite+ (v.2.4.0.3), Geomagic Wrap (v. 2021.0.0), DigTracePro (v. 1.8.1) |

For manuscripts utilizing custom algorithms or software that are central to the research but not yet described in published literature, software must be made available to editors and reviewers. We strongly encourage code deposition in a community repository (e.g. GitHub). See the Nature Portfolio guidelines for submitting code & software for further information.

## Data

Policy information about availability of data

All manuscripts must include a data availability statement. This statement should provide the following information, where applicable:

- Accession codes, unique identifiers, or web links for publicly available datasets
- A description of any restrictions on data availability
- For clinical datasets or third party data, please ensure that the statement adheres to our policy

Previously published data was obtained from ref.4,19-21,28,32,42. The authors declare that all other data supporting the findings of this study are available within the paper [and its supplementary information files], including original source data for figures [1-3], extended data figures [3-6], and extend data table [1]. The photogrammetric reconstruction of the Laetoli A trackway based on three original photographs from the 1977 expedition is available on Morphosource (accession #: in process). The photogrammetric reconstruction of Laetoli A trackway using 57 photographs taken of the re-excavated discovered Laetoli Site A footprints in 2019 is available on Morphosource (accession #: in process).

# Field-specific reporting

Please select the one below that is the best fit for your research. If you are not sure, read the appropriate sections before making your selection.

☐ Life sciences ☐ Behavioural & social sciences ☒ Ecological, evolutionary & environmental sciences

For a reference copy of the document with all sections, see nature.com/documents/nr-reporting-summary-flat.pdf

# Ecological, evolutionary & environmental sciences study design

All studies must disclose on these points even when the disclosure is negative.

**Study description**

This study compared footprint characteristics of black bears (Ursus americanus), chimpanzees (Pan troglodytes), and the fossilized footprints at Engare Sero, Tanzania with previously collected data on the footprints of Pan troglodytes and humans (Homo sapiens) to understand the fossilized footprints from Laetoli, Tanzania.

**Research sample**

The research sample at the Kilham Bear Center consisted of two groups. The first was a set of wild Ursus americanus from the local population in New Hampshire, US that were habituated to allow observation and video recordings. The second was a set of four semi-wild juvenile Ursus americanus (between the ages of 5-8 months). These individuals were chosen to represent the wild black bear population. These juvenile bears were rescued from the wild after being orphaned. These bears are returned to the wild once they reached a sufficient age to survive on their own. These juvenile bears were chosen given their similarity in foot length to the Laetoli Site A trackway. The research sample at Ngamba Island Chimpanzee Sanctuary included the 46 (n=18 male, 28 female; ages 12-36) adult semi-wild Pan troglodytes living at the sanctuary. This population is made up of individuals rescued from the wild due to varying circumstances (e.g., rescued from illegal pet or bushmeat trade). Semi-wild individuals were utilized in this study to avoid some of the changes in behavior and locomotion often present in zoo animals, while still presenting a feasible location for data collection. Data were taken from two published sources examining the bipedal footprint characteristics of two captive subadult male (between the ages of 6.5-7 years old) chimpanzees (Pan troglodytes) housed at Stony Brook University. These data came from Hatala et al., 2016a and Thompson et al., 2018. These 2 sets of chimpanzees were chosen to represent the wild chimpanzee population. The research sample from Engare Sero, Tanzania included a set of 113 footprints (age and sex unknown) belonging to Late Pleistocene Homo sapiens taken from an orthophoto of the site. Footprint characteristics represent fossilized footprints from an unshod/minimally shod population of humans made in similar circumstances to the Laetoli A site. Data were taken on shod human footprint characteristics recorded at the Boston Museum of Science from DeSilva and Gill, 2013. These data included convenience sample of adults (n=581, age 8-80; 366 female, 215 male) and children (n=73, age 2-7; 29 female, 44 male). Cross-stepping data were recorded on 10 adult shod humans (individuals between 19 and 48 years old, with 6 female, 3 male, and 1 non-binary represented) at Chatham University. Data from humans were chosen to represent the broader Homo sapiens population. Data on unshod/minimally shod humans [n=29 adults (15 male, 14 female, aged 18-47) and n=12 juveniles (10 male, 2 female, aged 4-15)] and the Laetoli G footprints (n=5) were taken from Hatala et al., 2013 and Hatala et al., 2016b. Data on the Laetoli S footprints were taken from Pelissero, 2017. The research sample from the Laetoli Site A, included the set of five preserved fossilized bipedal footprints.

**Sampling strategy**

Sample sizes for animal fieldwork were chosen based on the availability of individuals at semi-wild animal sanctuaries. Very little kinematic data exists for wild/semi-wild individuals due to the difficulties of collecting data in the field. The study recruited all individuals available at both the Kilham Bear Center and Ngamba Island Chimpanzee Sanctuary. Sample sizes for fossil footprint trackways (Laetoli A, S, and G) as well as Engare sero were determined based on the preservation of the footprints and availability of data. All viable, undistorted prints were included. For the novel human cross-stepping data, a sample size of 10 subjects producing 10 footprints each was sufficient for a quantitative assessment of how cross-stepping mechanics tended to influence both the perimeter dimensions and the internal topography of the tracks they produced.

**Data collection**

Video data on wild Ursus americanus behavior was recorded by B.H over the course of several years at his ongoing field site in Lyme, NH. These videos were digitized and analyzed by C.J. Data were collected on the juvenile black bears by E.M. and P.K. The juvenile bears were incited to walk independently bipedal across a mud trackway for either a maple syrup or applesauce reward. The mud trackway was then removed from the enclosure and the defined footprint characteristics were recorded. Data were collected on adult Pan troglodytes individuals at the the Ngamba Island Chimpanzee Sanctuary by E.M. Individuals walked across a plantar pressure mat placed in the walk way connecting their overnight enclosure and the open forest habitat. Individuals passed over the mat twice a day as the entered and exited the forest. Data on the Engare Sero footprint trackways were collected by C.L.-P., B.Z., and E.K. An orthophoto was generated of the site by by C.L.-P. and B.Z. Footprint characteristics were then measured on this orthophoto by E.K. using ImageJ. Data were collected on the Laetoli Site A prints by A.S.D., K.F., L.D.F., J.G., E.G., D.K., B.M., A.P., S.R., R.T., C.M.M., and J.M.D. who relocated, re-excavated, and measured the footprint characteristics of the original Site A trackway. Data were collected by K.H. and E.M.W on habitually shod humans walking with both their normal gait and a cross-stepping gait. The defined footprint characteristics were recorded and analyzed.

**Timing and spatial scale**

Data on the semi-wild chimpanzee at Ngamba Island Chimpanzee Sanctuary were recorded between December 9-16, 2018. Length of sampling was determined by funding and availability of the sanctuary. Data were collected twice a day; once in the morning (between 6:45-8:00 am) when the chimpanzees were headed to the forest for their first feeding, and once at 6:00 pm when the chimpanzees were headed into the overnight enclosure to sleep and receive their last feeding. Data on the juvenile bears at the Kilham Bear Center were recorded over the course of four visits in 2017 (May 11, Aug. 11, Aug. 14, Aug.31st). Length of sampling was determined by availability of researcher and sanctuary. Data were recorded in the morning/early afternoon for each visit so that data collection could occur prior to their feeding. This was to encourage participation as food was used as a reward. The Engare Sero orthograph was constructed using photographs taken between June 2-17, 2010. Multiple days were necessary to construct this composite image due to its size and variation in weather (i.e., some days were too windy to keep the tent over the camera. Data analysis on the Engare Sero footprints were collected and analyzed periodically between September 2019 and April 2020 as the work was being completed as part of E.K.'s graduate research. Data on the Laetoli Site A trackway were collected between June 19-25th,

| | |
|---|---|
| | 2019. Field season length was determined by funding and availability of researchers. Cross-stepping data were recorded between June 12-22, 2021 as that was sufficient to complete collection across the 10 participants. |
| Data exclusions | Footprint data were only excluded from the study if there was insufficient preservation of the track to measure the characteristics of interest defined in the methods. (E.g., a footprint might be excluded if it does not preserve the heel impression and thus cannot have the foot length measured). |
| Reproducibility | Footprint characteristics were defined prior to the start of the study. All novel footprints were preserved (including plaster casts of bear prints, 3D surface models of Laetoli Site A, the original in situ Site A prints, and plantar pressure impressions of Pan troglodytes) so measurements can be repeated by other researches. 3D surface scans of both the 1977 excavation and our 2019 re-excavation are publicly available on Morphosource for other researchers to access and validate our measurements. Reproducibility for individual footprints across our comparative sample (humans, bears, and chimpanzees) was established by collecting and comparing multiple footprints of the same individuals. |
| Randomization | Randomization was not relevant to our study as we were interested in measuring footprint characteristics from whole sample populations, as opposed to comparisons within those populations. |
| Blinding | Blinding was not relevant to the data collected on the non-human comparative species (e.g., bears and chimpanzees) nor to the data collection on fossilized footprints. The human participants were unaware of the site A tracks at Laetoli and therefore had no knowledge of how the data obtained from their footprints would be used in this study. |

Did the study involve field work? ☒ Yes ☐ No

## Field work, collection and transport

| | |
|---|---|
| Field conditions | Fieldwork was completed at two semi-wild animal sanctuaries, one in Entebbe, Uganda and one in New Hampshire, US. Additional fieldwork was conducted at Laetoli, Tanzania. For fieldwork completed at both animal sanctuaries, environmental conditions were unlikely to effect the outcome of the experiment. For both locations, data collection occurred during clear weather at temperatures that were within seasonal norms. For the fieldwork at Laetoli, the weather was clear and within seasonal norms and thus unlikely to have impacted data collection. |
| Location | Fieldwork was conducted at three locations: 1) the fossil site of Laetoli, Tanzania (S 03.13.185' E 035.11.976' taken on-site with Garmin GPS) during June 2019; 2) Ngamba Island Chimpanzee Sanctuary located on chimpanzee island (-0.10409381597120933, 32.652780666056096 from Google Map) near in Entebbe, Uganda in December 2018; and 3) the Kilham Bear Center (43.770876615493975, -72.09782685342287 from Google Map) in Lyme, NH in 2017. |
| Access & import/export | Fieldwork was conducted at several locations. Research permits were granted by the Tanzanian Commission for Science and Technology (permit 2019-370-NA-2019-2016) to access the site of Laetoli in Tanzania. Permission to study the chimpanzees in Uganda was provided by the Uganda Wildlife Authority (UWA/COD/96/05) and the Ugandan National Council for Science and Technology (NS65ES). No materials were imported/exported. Permission to study the black bears at Kilham Bear Center was provided by the sanctuary director, B. Kilham. No animal materials were exported/imported. All animal protocols were approved by the Dartmouth College Institutional Animal Care and Use Committee. |
| Disturbance | The study exposed the surface of the fossilized footprints at Site A in Laetoli, Tanzania by disturbing the top soil covering this trackway. This disturbance was minimized by the careful reburial of the trackway to prevent potential damage to the footprints. The study caused a slight disturbance to the daily routine of the chimpanzees at Ngamba Island Chimpanzee sanctuary and the black bears at the Kilham Bear Center. This disturbance was minimized through study design input by the Sanctuary and Center staff as well as efforts to habituate the individuals to the presence of the research and plantar pressure mat. |

# Reporting for specific materials, systems and methods

We require information from authors about some types of materials, experimental systems and methods used in many studies. Here, indicate whether each material, system or method listed is relevant to your study. If you are not sure if a list item applies to your research, read the appropriate section before selecting a response.

### Materials & experimental systems

| n/a | Involved in the study | |
|---|---|---|
| ☒ | ☐ | Antibodies |
| ☒ | ☐ | Eukaryotic cell lines |
| ☐ | ☒ | Palaeontology and archaeology |
| ☐ | ☒ | Animals and other organisms |
| ☐ | ☒ | Human research participants |
| ☒ | ☐ | Clinical data |
| ☒ | ☐ | Dual use research of concern |

### Methods

| n/a | Involved in the study | |
|---|---|---|
| ☒ | ☐ | ChIP-seq |
| ☒ | ☐ | Flow cytometry |
| ☒ | ☐ | MRI-based neuroimaging |

## Palaeontology and Archaeology

| | |
|---|---|
| Specimen provenance | We received approval from the Tanzania Commission for Science and Technology (permit 2019-370-NA-2019-2016) to conduct |

| | |
|---|---|
| Specimen provenance | research at site of Laetoli. |
| Specimen deposition | The fossil footprints remain in situ at site A in locality 7 at Laetoli, Tanzania. The Site A footprint excavation has been reburied to protect the footprints and the site marked. |
| Dating methods | no new dates are provided |

☐ Tick this box to confirm that the raw and calibrated dates are available in the paper or in Supplementary Information.

| | |
|---|---|
| Ethics oversight | Ethical oversight of the research project was provided by the Tanzania Commission for Science and Technology. |

Note that full information on the approval of the study protocol must also be provided in the manuscript.

## Animals and other organisms

Policy information about studies involving animals; ARRIVE guidelines recommended for reporting animal research

| | |
|---|---|
| Laboratory animals | The study did not involve laboratory animals |
| Wild animals | Study examined four semi-wild juvenile black bears (Ursus americanus) housed at the Kilham Bear Center in Lyme, NH awaiting reintroduction into the wild. These individuals (3 male, 1 female) were released back into the wild (Vermont and New Hampshire) after study completion through cooperation between the Kilham Bear Center and New Hampshire Fish and Game Department. The study followed the bears between the age of 5 to 8 months old. Additionally, this study examined just over 50 hours of video data recording Ursus americanus behavior on wild black bears in New Hampshire, US. These included both adult and juvenile individuals (precise age and gender unknown). Study also examined 46 adult (n=18 male, 28 female; ages 12-36) semi-wild chimpanzee (Pan troglodytes) individuals housed at the Ngamba Island Chimpanzee Sanctuary in Entebbe, Uganda. These individuals remain at the Ngamba Island Chimpanzee Sanctuary. No individuals were captured in the field or transported for the study. |
| Field-collected samples | The study did not involve samples collected in the field. |
| Ethics oversight | Ethical approval was provided by the Dartmouth College Institutional Animal Care and Use Committee. |

Note that full information on the approval of the study protocol must also be provided in the manuscript.

## Human research participants

Policy information about studies involving human research participants

| | |
|---|---|
| Population characteristics | Data were collected from 10 healthy adults (including 6 female, 3 male, and 1 non-binary between 19 and 52 years old) without foot or other lower limb maladies that might affect their mobility. Neither age nor gender was expected to affect the hypotheses being tested (how footprint morphology changes due to cross-stepping). |
| Recruitment | Participants were recruited via email and by word-of-mouth (snowball sampling). We did not identify the potential for any form of recruitment bias that may impact results. |
| Ethics oversight | Ethical approval was provided by the Chatham University Institutional Review Board |

Note that full information on the approval of the study protocol must also be provided in the manuscript.
